# Facile Synthesis of Thermoresponsive Alternating Copolymers with Tunable Phase-Transition Temperatures

**DOI:** 10.3390/polym16243470

**Published:** 2024-12-12

**Authors:** Zichen Huang, Fan Chen, Qi Wang, Dingxiang Zhang, Hongdong Wang, Xiacong Zhang

**Affiliations:** 1 Department of Polymer Materials, School of Materials Science and Engineering, Shanghai University, Shanghai 200444, Chinazb933@shu.edu.cn (F.C.);; 2 School of Mechatronic Engineering and Automation, Shanghai University, Shanghai 200444, China

**Keywords:** thermoresponsive polymers, amphiphilic copolymers, borate ester, polyethylene glycol

## Abstract

A series of novel amphiphilic alternating CPEG copolymers were synthesized through an amine–epoxy click reaction comprising aliphatic amine and polyethylene glycol diglycidyl ether (PEGDE). These polymers were characterized in detail via nuclear magnetic resonance (NMR), gel permeation chromatography (GPC), Fourier-transform infrared spectroscopy (FTIR), and thermogravimetric analysis (TGA) to confirm the successful synthesis. Due to their amphiphilic structure, these polymers display thermoresponsiveness, with tunable cloud points (Tcps) that are adjustable from 20.8 °C to 46.8 °C by altering the side-chain length of the aliphatic amine, varying the mixing ratios of copolymers, the solution’s pH, and salt additions. This tunable thermoresponsive behavior positions CPEG copolymers as promising candidates for a range of functional material applications.

## 1. Instruction

Stimuli-responsive polymers are able to respond to changes in the external environment, such as temperature [1,2,3], light [4,5,6], pH [7,8], mechanical forces [9], and chemical stimuli. They can change their physical and/or chemical properties reversibly or irreversibly. These polymers undergo reversible or irreversible changes in their physical and/or chemical properties, making them invaluable for applications in drug delivery [10,11], biosensors [12], diagnostics, and separation technologies [13]. Among them, thermoresponsive polymers, which exhibit reversible phase-transitions triggered by temperature changes, form one of the most prominent types due to their unique properties and broad biomedical applications, including bioimaging [14], drug delivery [15,16], injectables [17,18], smart surfaces [19,20,21,22], adhesives [23], and tissue engineering [24,25,26]. The most common thermoresponsive polymers, such as poly(*N*-alkylacrylamides), are poly(*N*-isopropylacrylamide) (PNIPAM) and the polyethylene glycol (PEG) derivative OEGMA [27]. PNIPAM, for instance, undergoes a pronounced phase-transition upon heating while showing significant hysteresis during cooling due to both intramolecular and intermolecular hydrogen bonding. Lutz [28] investigated the lower critical solution temperature (LCST) of poly(2-(2-methoxyethoxy) ethylmethacrylate-*co*-(ethylene glycol) methacrylate) (P(MEO_2_MA-*co*-OEGMA_475_)) copolymers, which exhibited similar or superior temperature-sensitive transition properties in comparison to PNIPAM, with added benefits for biological applications due to reduced toxicity. Polymers with LCSTs or cloud point temperatures (Tcps) in aqueous solutions are of particular interest. When heated above their Tcps, these polymers undergo entropy-driven dehydration, causing polymer chain collapse and aggregation into mesoglobules. One critical characteristic of thermoresponsive polymers is the tunability of their Tcps. Tcp is primarily governed by the hydrophilic–hydrophobic balance within the polymer structure, with higher hydrophilicity generally increasing Tcp. The thermoresponsive behavior of block copolymers is typically determined by their thermoresponsive blocks, while random copolymers’ Tcps depend on the overall hydrophobicity of the entire polymer. By adjusting the ratio of hydrophilic and hydrophobic units, the Tcps of copolymers can be tuned accordingly. However, most thermoresponsive polymers are covalently linked, meaning that making post-synthesis structural adjustments to finely tune their LCSTs without breaking the polymer’s covalent bonds is challenging. Therefore, it remains an important challenge to develop novel strategies such as supramolecular interactions [29] and dynamic covalent chemistry [30] for preparing thermoresponsive polymers with truly tunable Tcps.

Polyethylene glycol diglycidyl ether (PEGDE), with its α, ω-epoxy terminal groups, is a highly reactive cross-linker commonly used in hydrogel fabrication due to its ability to undergo crosslinking reactions. For that reason, PEGDE is always used as a cross-linker in hydrogel fabrication. It has been proven that when this polymer is subcutaneously injected at lower doses, it is non-poisonous [31]. Meanwhile, injectable hyaluronic acid (HA)-based hydrogels mainly comprising PEGDE are presently used as the new standard for soft-tissue fillers [32]. The formation of a PEGDE polymer network can be used effectively for improving the mechanical strength of solid electrolytes in Li-ion batteries [33]. PEGDE can also be used to produce microelectrode biosensors [34]. Therefore, PEGDE demonstrates significant potential across various fields, from biomedical applications to energy storage applications.

In our previous study, a series of hydrogels were synthesized through a cross-linking process involving CPEG polymers and alginate grafted with phenylboronic acid (Alg-PBA) [35]. CPEG polymers, obtained by reacting PEGDE with pentylamine, exhibited amphiphilic structures and demonstrated thermoresponsive behavior in aqueous solutions. Building on this foundation, the current study aims to investigate the thermoresponsive properties of a series of alternating copolymers with tunable hydrophilic and hydrophobic segments. These copolymers were synthesized via condensation polymerization between different primary amines and PEGDE. Their thermoresponsive behavior was characterized via UV–vis spectroscopy in order to determine their Tcps under different conditions, including variations in the aliphatic amine side-chain length, mixing ratios of copolymers, solution pH, and salt concentration. In addition, their self-assembly behavior was thoroughly examined using dynamic light scattering (DLS), allowing for detailed exploration of the mechanisms driving their thermoresponsiveness. Notably, the reactive diethanolamine (DEA) units in the polymer backbone enable the formation of borate esters upon reaction with boronic acid, and this is stabilized via intramolecular N-B coordination. The introduction of phenylboronic acid derivatives further allowed the fine-tuning of Tcp, expanding the versatility of these materials. This research advanced our understanding of CPEG’s thermoresponsive behavior and expanded their potential applications in responsive material design.

## 2. Materials and Methods

### 2.1. Materials

Poly(ethylene glycol) diglycidyl ether (PEGDE; average molecular weight = 500) was purchased from Aladdin (Shanghai, China). The *n*-pentylamine (C5-NH_2_, >98%) and *n*-hexylamine (C6-NH_2_ > 98%) were purchased from TCI (Tokyo, Japan). The *n*-butylamine (C4-NH_2_, >98%) was purchased from Adamas-beta (Shanghai, China). All other chemicals were used without further purification.

### 2.2. Synthesis of CPEG

C5PEG was synthesized from C5-NH_2_ and PEGDE as monomers via the amine–epoxy click reaction. Generally, PEGDE (2 g, 3.8 mmol) and C_5_-NH_2_ (480 μL, 3.8 mmol) were added into a 10 mL reaction tube. The super-dry reagent of DMF was also added as a solvent. The air in the tube should be replaced by N_2_. After stirring for 12 h at 60 °C, dimethylformamide (DMF) was removed by rotary evaporation. The polymer was dissolved in dichloromethane (DCM) and extracted with a 10 wt% NaOH aqueous solution. Using rotary evaporation to remove DCM, the colorless viscous liquid was obtained. C4PEG and C6PEG were synthesized in a similar manner. The only difference was that C_4_-NH_2_ and C_6_-NH_2_ were applied as monomers. C4-C6PEG was synthesized from C4-NH_2_ (240 μL, 1.9 mmol), C6-NH_2_ (240 μL, 1.9 mmol), and PEGDE (2 g, 3.8 mmol) as monomers via the same solution and processes as C5PEG.

### 2.3. Characterization of Synthesized Polymers

^1^H NMR spectra of C6PEG, C5PEG, and C4PEG were recorded on an NMR spectrometer (AV-500; Bruker, Zurich, Switzerland) using CDCl_3_ as the solvent. Fourier-transform infrared (FTIR) spectroscopies of C6PEG, C5PEG, and C4PEG were obtained using an FTIR spectrometer (Nicolet Is 50; Thermo Fisher Scientific, Waltham, MA, USA). The number-average molecular weight (Mn), weight-average molecular weight (Mw), and polydispersity index (Ðs) of the synthesized C6PEG, C5PEG, and C4PEG were characterized via gel permeation chromatography (GPC) using a gel permeation chromatography system (Breeze; Waters Corporation, Milford, CT, USA) at a test concentration of 2 mg⋅mL^−1^ with DMF as the eluent. Thermogravimetric analysis (TGA) was carried out using a PerkinElmer Q5000IR thermobalance, and nitrogen was used as the purging gas at a heating rate of 20 °C min^−1^. The liquid samples were around 5 mg. The DSC measurement was carried out on a Thermal Advantage DSC (DSC 25; TA Instruments, New Castle, DE, USA) equipped with a refrigerator cooling system. The polymer (10 mg) was placed in aluminum pans (non-hermetic) and scanned at a heating rate of 10 °C min^−1^ from −80 to 100 °C under a dry nitrogen atmosphere.

### 2.4. Characterization of the Self-Assembly Process of Polymer Micelles

The aggregate sizes of C6PEG, C5PEG, and C4PEG micelles were measured using a dynamic light scattering (DLS) instrument (DynaPro NanoStar M3300; Wyatt, Santa Barbara, CA, USA). Prior to measurement, the solutions were filtered through a 0.45 μm filter to remove dust. The hydrodynamic radii of aggregates were calculated following the Stokes–Einstein equation.

The critical micelle concentrations (CMC) of C6PEG, C5PEG, and C4PEG in water were measured using 1,6-diphenyl-1,3,5-hexatriene (DPH) as a hydrophobic dye probe. Solutions of C6PEG, C5PEG, and C4PEG at varying concentrations (0.01, 0.1, 0.5, 1, 3, and 5 wt%) were prepared, and 4 μM DPH was added into each solution. These solutions were equilibrated in the dark for 4 h before their absorbance from 300 to 450 nm was measured via a UV–vis spectrophotometer (V750; JASCO, Hachioji, Tokyo, Japan). The inflection points of the absorbance difference between 377 and 400 nm against the logarithm concentration of the copolymer solution were equal to the CMC value [35].

### 2.5. Characterization of Factors of Polymer Phase-Transition Temperature

A UV–vis spectrophotometer equipped with a thermo-controlled bath (V750; JASCO, Hachioji, Tokyo, Japan) was utilized to analyze the transmittance variations of CPEG solutions with different salts, pHs, and phenylboronic acid derivatives. The heating rate was set at 0.5 °C⋅min^−1^. Measurements were performed using a light-source wavelength of 700 nm, and absorbance was recorded every 5 s.

## 3. Result and Discussion

### 3.1. Synthesis and Characterizations of Copolymers

**Figure 1:**
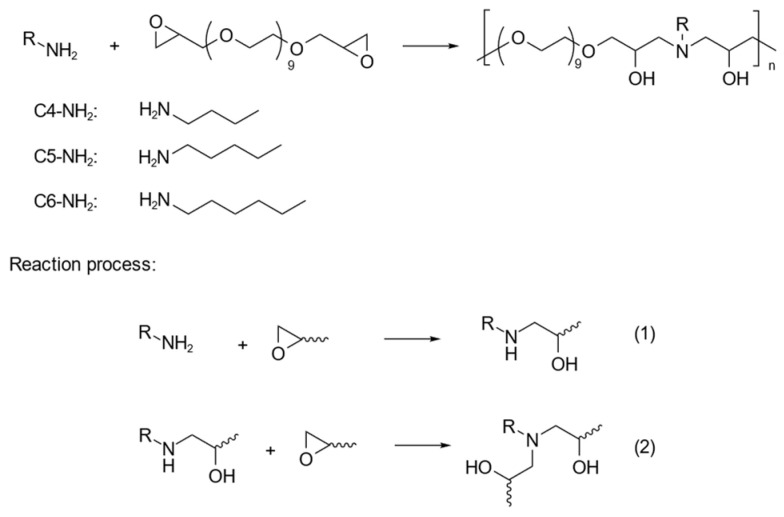
Synthesis scheme of C4PEG, C5PEG, and C6PEG polymers.

The amphiphilic alternating copolymers, CPEG, were synthesized via amine–epoxy condensation copolymerization between the PEGDE and alkylamines. The structural formulas are presented in Figure 1. The copolymerization process involved the reaction of primary amine groups with the epoxy groups under DMF as the solvent at 60 °C. This amine–epoxy condensation resulted in the formation of secondary amines as intermediates. These secondary amines could further react with additional epoxy groups, facilitating the growth of the polymer chain via condensation polymerization. Three different alkylamines—*n*-butylamine, *n*-pentylamine, and *n*-hexylamine—were used to synthesize the copolymers C4PEG, C5PEG, and C6PEG, respectively. The successful polymerization resulted in transparent, viscous liquid products once the solvent was removed, with yields exceeding 82% for all three polymers.

**Figure 2:**
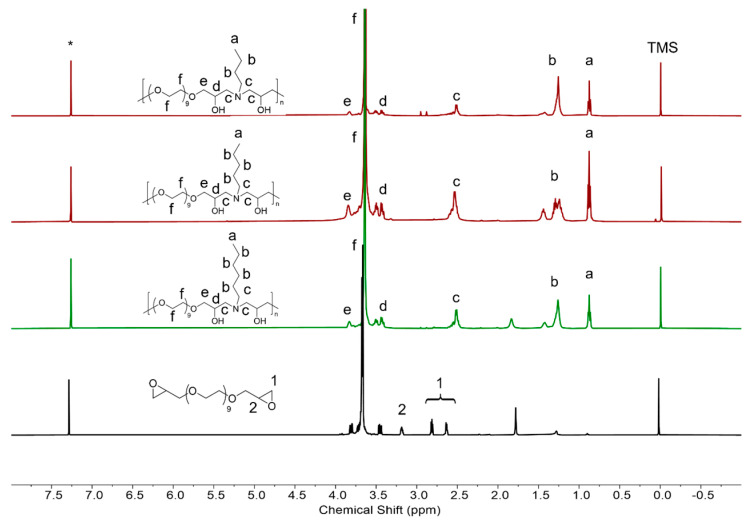
^1^H NMR spectra of C4PEG, C5PEG, C6PEG, and PEGDE in CDCl_3_. Chemical shifts are reported as *δ* values (ppm) relative to internal Me_4_Si (TMS). Solvent signals are marked with *.

The chemical structures of the CPEG copolymers were characterized via ^1^HNMR, as shown in Figure 2. Peak a at 0.88 ppm represented the terminal methyl group of the alkyl side chain. The chemical shifts of methylene groups located between the initial and terminal methyl groups of the alkyl side chain were in the range of 1.3–1.4 ppm (b). The difference in Peak b exhibited the difference in alkyl side chains. Peak c represented the initial methylene of the alkyl side chain, which was linked with the nitrogen atom, and their chemical shifts changed to 2.5 ppm after the amine–epoxy click reaction due to nitrogen effects. Peak f belonged to the polyethylene glycol (PEG) chain. The disappearance of the peaks in PEGDE refers to the epoxy groups, which were originally at 2.5–2.8 (Peak 1) and 3.3 ppm (Peak 2), respectively. The appearance of peaks at 3.5 ppm (d) and 2.5 ppm (c), along with the disappearance of Peaks 1 and 2, indicates that the reaction between the epoxy and the primary amine was successful.

**Figure 3:**
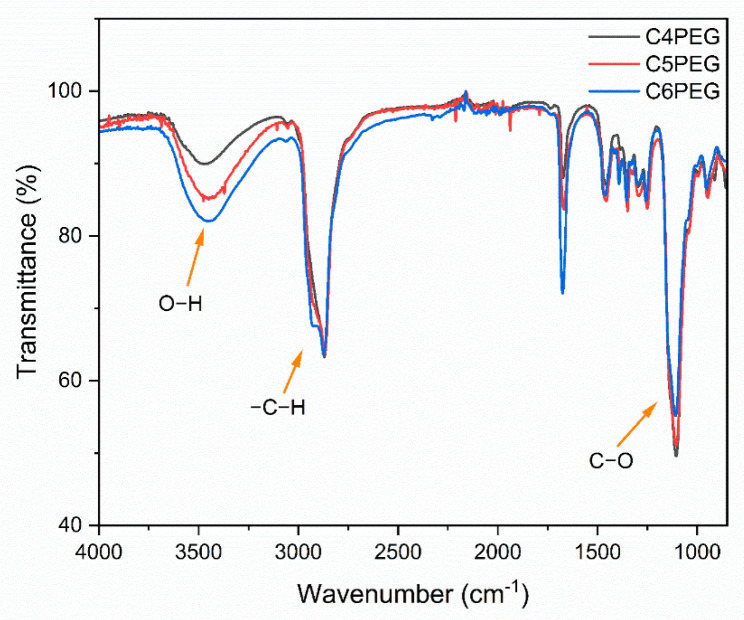
ATR-FTIR spectra of C4PEG, C5PEG, and C6PEG.

The molecular structures of CPEG were further confirmed via attenuated total reflection Fourier-transform infrared (ATR-FTIR) spectroscopy, as presented in Figure 3. The broad absorption band observed between 3300 and 3600 cm^−1^ corresponded to hydroxyl (O−H) stretching vibrations, which are indicative of the presence of hydroxy groups in the polymer chains. Additionally, a broad peak located at approximately 2860 cm^−1^ was associated with the stretching vibration of alkyl (−C−H) bonds, which are typical of the polymer backbone. The intense band appearing at around 1098 cm^−1^ was attributed to C−O stretching vibrations, confirming the presence of ether groups in the molecular structure. These characteristic peaks suggested the successful synthesis of CPEG variants, with consistent molecular structures across different chain lengths (C4PEG, C5PEG, and C6PEG).

The average molecular weights of the CPEG polymers were measured using gel permeation chromatography (GPC), and the resulting GPC curves are presented in Figure S1. The presence of shoulders in the GPC analysis is due to oligomers. The Ðs for the three CPEG samples were approximately 1.3, indicating relatively narrow molecular weight distributions. This was due to the low degree of polymerization with respect to CPEG removal during the alkaline washing process. The molecular weights of the CPEG polymers were found to be quite similar, with number-average molecular weights (Mn) ranging from 6.6 × 10^^3^^ to 7.2 × 10^^3^^ and weight-average molecular weights (Mw) ranging from 8.3 × 10^^3^^ to 9.2 × 10^^3^^. However, the degree of polymerization for the CPEGs synthesized using this method was relatively low, at approximately 16–18. This lower degree of polymerization could be attributed to the reduced reactivity of the secondary amine in the reaction, as well as potential inaccuracies in the proportion of raw materials used during the synthesis. To increase the degree of polymerization, an alternative approach was explored by synthesizing C5PEG without solvents. Since both PEGDE and the amine were liquids, bulk polymerization was considered feasible, resulting in an increase in monomer concentration. As observed in the data shown in Table 1, this solvent-free method successfully increased the average molecular weight of the C5PEG polymer. The Mn increased to 13.0 × 10^^3^^, and the Mw increased to 26.4 × 10^^3^^. However, this method also resulted in an increase in polydispersity, which rose to 2.0.

**Figure 4:**
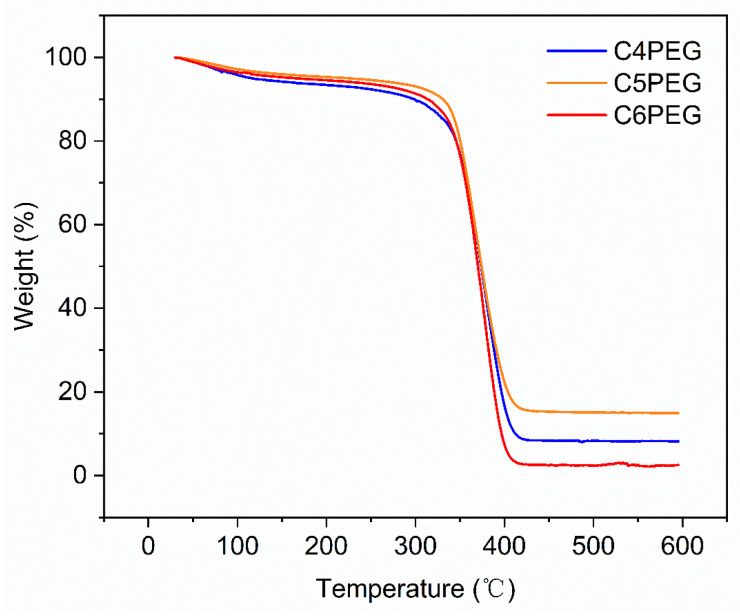
The TGA curves of C4PEG, C5PEG, and C6PEG.

**Figure 5:**
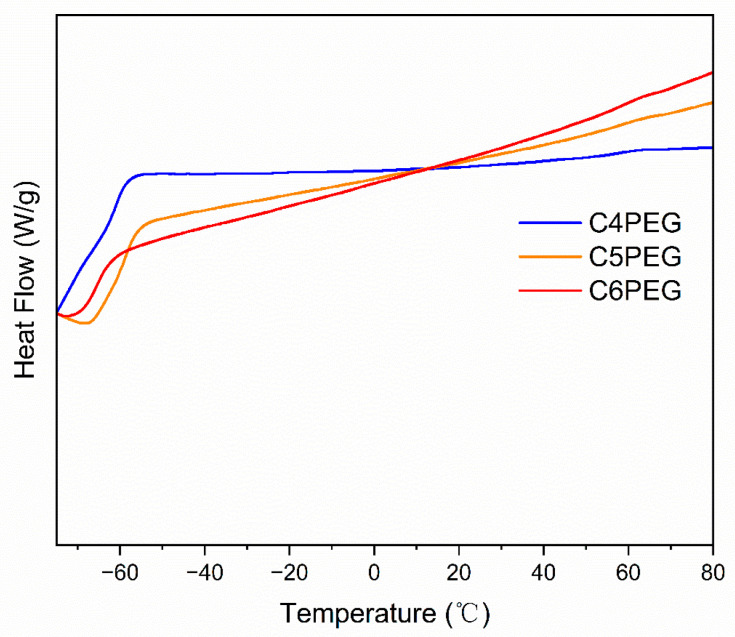
The DSC curves of C4PEG, C5PEG, and C6PEG.

The thermal properties of CPEG copolymers were evaluated via TGA and DSC, as shown in Figure 4 and Figure 5. The results demonstrated that these series of copolymers exhibit similar thermal stability. The initial decomposition temperature of C4PEG was 295.0 °C, while C5PEG and C6PEG display decomposition temperatures of 329.7 and 312.0 °C, respectively. The glass transition temperatures of C4PEG, C5PEG, and C6PEG were −64.84 °C −60.46 °C, and −65.24 °C, respectively. Interestingly, thermal stability and glass transition temperature generally increased with the length of the side group, with C5PEG being an exception to this trend. This anomaly could be attributed to the distinct arrangement of the odd-numbered carbon chains in C5PEG compared to the even-numbered carbon chains in C4PEG and C6PEG, which likely affected molecular packing and thermal behavior. The even number for the carbon chain was associated with larger average molecular interchain spacings. The increase in the average molecular interchain spacing value resulted in a decrease in thermal conductivity. This was the reason why C5PEG exhibited a better thermal stability and glass transition temperature [36].

### 3.2. Thermoresponsive Behaviors of Copolymers

**Figure 6:**
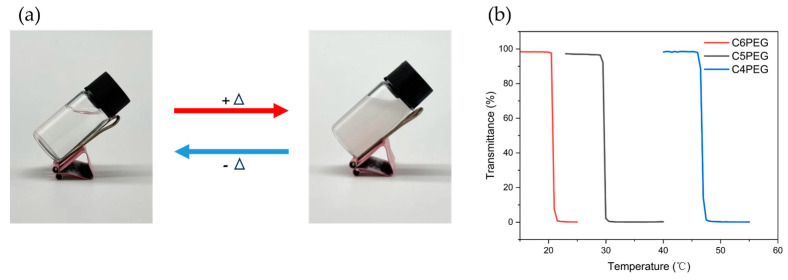
(**a**) Photographs of an aqueous solution of C5PEG at 20 °C (**left**) and 50 °C (**right**). (**b**) The curves of transmittance versus temperature for C4PEG, C5PEG, and C6PEG in the 5 wt% aqueous solution.

The structural characteristics of the polymers were determined by alternating the arrangement of PEGDE and alkylamine units. The variation in alkyl chain length from butyl (C4) and amyl (C5) to hexyl (C6) introduced hydrophobicity gradients along the polymer chain. This amphiphilic nature of the copolymers was expected to influence their self-assembly and thermoresponsive behavior. The thermoresponsiveness of the CPEG aqueous solution is shown in Figure 6a. When the temperature was below the transition temperature, the C5PEG aqueous solution remained transparent. It was found that the thermoresponsiveness of all samples was reversible, and the solutions returned from turbid to transparent upon the cooling of the systems. Temperature-varied UV−vis spectroscopy was used to measure Tcps. Tcp is defined as the temperature corresponding to the 50% transmittance point. The transmittance of each CPEG solution decreased rapidly after its temperature reached a point above the transition temperature, as shown in Figure 6b. The Tcps of C4PEG, C5PEG, and C6PEG were 46.8, 29.7, and 20.8 °C, respectively, demonstrating that their Tcps would decrease as the lengths of side chain increased. This was attributed to the enhanced hydrophobicity of the CPEG polymer, which was triggered by the longer side alkyl chains. The increase in the hydrophobicity of the alkyl units might have an influence on the self-assembly and aggregation of polymers in water. Accordingly, C6PEG in the aqueous solution was turbid at room temperature, while C4PEG in the aqueous solution with higher hydrophilicity was transparent at the same temperature.

**Figure 7:**
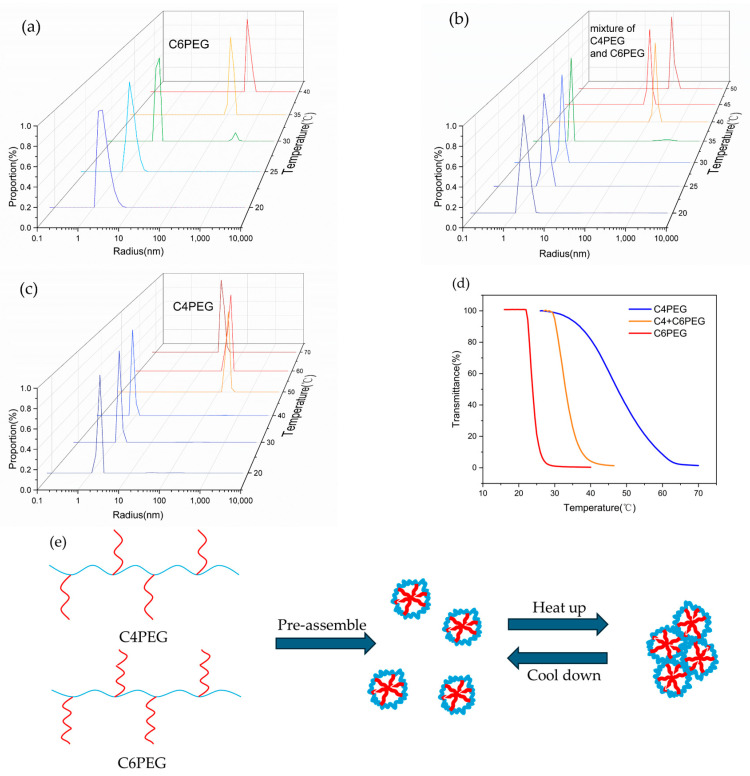
Dependence of the hydrodynamic radius (*R*_h_) of (**a**) C6PEG, (**b**) C4PEG, and (**c**) the mixture of C4PEG and C6PEG in aqueous solutions on temperature. (**d**) Transition temperatures of C4PEG, C6PEG, and the mixture of C4PEG and C6PEG at 0.5 wt% concentration. (**e**) Schematic diagram of the thermally induced aggregation behaviors of the CPEG mixture.

C6PEG and C4PEG were chosen for further study; these had the lowest Tcp and highest Tcp among the CPEGs. The, in general, thermally induced aggregation behaviors of CPEGs are shown in the substantial differences in their Tcps. The thermally induced phase transition was thus studied via dynamic light scattering (DLS), and the aggregate sizes of the CPEG polymers were determined. The results are plotted in Figure 7 from sample C6PEG (a), which comprises a mixture of C4PEG and C6PEG (b), and C4PEG (c) with a diluted concentration (0.5 wt%). Lower concentrations were chosen to minimize multiple light scattering effects, ensuring that size measurements were more accurate [37]. As the temperature increased, the size of the copolymer particles increased from approximately 2 nm to above 200 nm, indicating a characteristic thermoresponsive aggregation behavior.

For the further study of the thermally induced aggregation behaviors of CPEGs, the critical micelle concentrations (CMCs) of the CPEGs were measured. To measure CMC, DPH hydrophobic dye was added to the polymer solution. The absorbance of DPH increased after the micelles were formed. The CMC of different polymers depended on the differences in absorbance between 377 and 400 nm against the logarithm of copolymer concentrations. The absorbance curves between 300 and 400 nm in the C4PEG and C6PEG solutions are shown in Figures S2 and S3, respectively. As shown in Figure S4, the CMC values of C6PEG, C5PEG, and C4PEG were 0.22 wt%, 0.40 wt%, and 0.65 wt%, respectively [35]. This variation in CMC values was attributed to differences in the hydrophobic alkyl chain length in the aliphatic amine segments of the copolymers. Longer alkyl chains, such as those in C6PEG, resulted in lower CMC values, while shorter chains, such as in C4PEG, resulted in higher CMC values. The CMC of C4PEG (0.65 wt%) was higher than the concentration used in the DLS experiments, indicating that C4PEG possibly dissolved single-chain conformations in water with a temperature of 20 °C. In contrast, the concentration of C6PEG in the DLS tests exceeded its CMC, providing evidence that 0.5 wt% C6PEG formed micelles at 20 °C. However, the particle size of C6PEG at 20 °C was similar to that of C4PEG, at approximately 2 nm, suggesting that C6PEG might have formed pre-assembled aggregates rather than fully developed micelles. The PDI values of C4PEG, the mixture of C4PEG and C6PEG, and C6PEG were 0.266, 0.436, and 0.565 under 20 °C, respectively. For C4PEG, the mixture of C4PEG and C6PEG, and C6PEG at 20 °C, the values were 0.266, 0.436, and 0.565, respectively. The higher PDI value of the mixture compared to individual copolymers suggested a broader size distribution, indicating the coexistence of different micelle populations. At 50 °C, the particle sizes of the mixture of C4PEG and C6PEG were comparable to those of the individual copolymers, further supporting the hypothesis that the two polymers formed micelles together. Additionally, both the PDI and particle size in the mixed solution at 50 °C were intermediate between the values for the pure copolymers, further confirming the co-assembly of C4PEG and C6PEG into micelles.

For comparison, the Tcp of C4PEG, C6PEG, and the mixture of C4PEG and C6PEG was tested at 0.5 wt% concentration via temperature-varied UV−vis spectroscopy. The Tcp of the C4PEG and C6PEG mixture was found to be intermediate relative to the individual transition temperatures of C4PEG and C6PEG, as shown in Figure 7d. This shift in Tcp, supported by dynamic light scattering (DLS) data, confirmed that C4PEG and C6PEG co-assembled into micellar structures. The hydrophobic alkyl chains of both polymers influenced the Tcp of the mixture. The thermally induced self-assembly process is illustrated in Figure 7e. At low temperatures, the polymer chains in the aqueous solution self-assembled into micelles, with hydrophilic PEG chains forming the exterior and hydrophobic alkyl side chains enclosed within the micelle core. As the temperature increased, micelles began to coalesce, driven by the dehydration of the hydrophilic domains. During this process, the polymer chains experienced a reduction in solvation, causing them to aggregate on the micelle surfaces. With a further increase in temperature, the micelles continued to grow, ultimately transitioning into larger particles at a radius of approximately 250 nm—nearly 200 times the original micelle size. This size increase resulted in visible aggregation in the aqueous solution, resulting in a sharp decrease in transmittance. This growth in particle size was characteristic of thermally induced aggregation, closely resembling behaviors observed in PNIPAM systems [38]. The hydrophobic interactions between the alkyl chains of C4PEG and C6PEG were crucial to this transition, as particle growth was directly linked to the temperature-induced collapse of hydrophilic domains and the subsequent aggregation of hydrophobic segments. These findings indicate that the Tcp of the CPEG polymer could be tuned by varying the mixing ratios of copolymers with different hydrophilicity conditions.

### 3.3. Methods for Tuning the Phase-Transition Temperature

**Figure 8:**
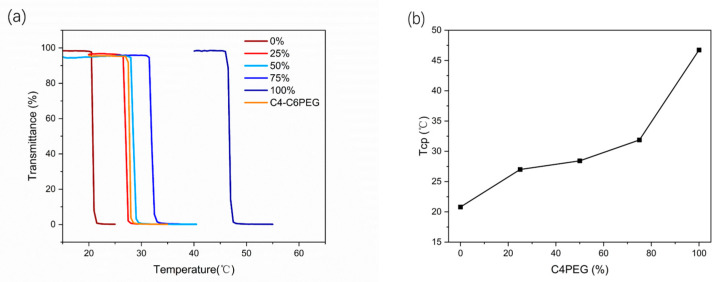
(**a**) Effects of different mixing ratios in C6PEG and C4PEG mixtures (copolymer concentration 2.5 wt%) on the change in cloud points; the proportion of C4PEG is shown in the figure. Comparison of the cloud points of C4PEG and C6PEG copolymers and the C4PEG and C6PEG mixture. (**b**) Tcp-dependence of the C4PEG and C6PEG mixture on the content of C4PEG.

To further explore tunability with respect to phase-transition temperatures (Tcp), the effects of C6PEG and C4PEG with varying mix ratios were studied at a total copolymer concentration of 2.5 wt% using temperature-dependent UV−vis spectroscopy. By examining different C4PEG and C6PEG ratios, the aim was to broaden the application potential of the system. Figure 8 illustrates the cloud point changes for mixtures with C4PEG contents at 0% (20.8 °C), 25% (26.9 °C), 50% (28.5 °C), 75% (31.9 °C), and 100% (46.8 °C). The results demonstrated, in a clear trend, that Tcp could be easily adjusted by altering the relative proportions of the two copolymers. The data indicated a decrease in Tcp by approximately 1.5 °C when the C4PEG content increased from 50% to 75%, with a smaller shift observed for pure C4PEG. This tunable Tcp range, spanning from 20.8 °C to 46.8 °C, encompassed both room temperature (25 °C) and body temperature (37 °C) contexts, and this holds significant promise for medical applications, including temperature-sensitive indicators and drug delivery systems.

For comparative analysis, a copolymer designated as C4-C6PEG was synthesized directly from poly(ethylene glycol) diglycidyl ether (PEGDE) and an equimolar mixture of n-butylamine and n-hexylamine. The Tcp of C4-C6PEG was found to be 27.7 °C, closely matching the Tcp of a 1:1 C4PEG and C6PEG mixture (28.5 °C). This similarity further underscored the robustness of the Tcp tuning achievable by blending, as comparable phase-transition temperatures could be obtained by either copolymerizing mixed amines or by physically blending distinct copolymers. These findings demonstrate that fine-tuning the phase-transition temperature through simple mixing strategies enables customized thermoresponsive behavior, making these copolymer systems adaptable for a range of biomedical and environmental applications.

With the exception of the changing proportions of the CPEG mixture, salt addition and pH regulation are common methods for changing phase-transition temperatures [39]. Given its moderate Tcp, C5PEG was selected as a model system to assess Tcp variations in response to different salts and pH. Previous studies have shown that salt can significantly influence the phase-transition behavior of thermoresponsive polymers, an effect often described via the Hofmeister (HS) series. While cations generally exert a minor influence on Tcp, according to the HS series, anions play a primary role. Therefore, sodium was used as the fixed cation, and anion types were varied among different salts. The effects of NaCl, Na_2_SO_4_, and NaPF_6_ on the thermally induced phase transition of C5PEG were investigated by adding these salts (50 mmol·L^−1^) to 2.5 wt% C5PEG solutions. The observed Tcp variations largely aligned with the HS series. For example, the addition of “salt-out” anions, such as Cl^−^ and SO_4_^2−^, lowered Tcp from 29.1 °C to 26.7 °C and 22.2 °C, respectively. In contrast, the “salt-in” anion PF_6_^−^ increased Tcp from 29.1 °C to 32.1 °C. Anions with lower lyotropic numbers tend to dehydrate the polymer chains by displacing water molecules, thus strengthening hydrophobic interactions and promoting polymer collapse into particle states. Conversely, anions with higher lyotropic numbers interfere with the structure of bulk water, enhancing polymer hydrophilicity [40].

**Figure 9:**
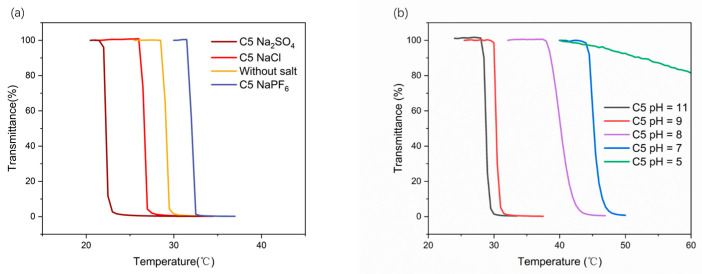
(**a**) Effects of different salt additions on the cloud point of the 2.5 wt% C5PEG aqueous solution. (**b**) Effects of different pH values on the turbidity point of the 2.5 wt% C5PEG aqueous solution.

Beyond salt addition, the solution’s pH also affected Tcp. As shown in Figure 9b, C5PEG solubility in water increased with increasing acidity. Tcp values at pH 11 and pH 9 were 28.8 °C and 30.3 °C, respectively, but increased to 40.2 °C and 45.2 °C at pH 8 and pH 7, respectively; moreover, it exceeded 80 °C at pH 5. In basic conditions, C5PEG remains largely insoluble due to the hydrophobic nature of its tertiary amine groups. However, in acidic conditions, the presence of H⁺ ions protonates the tertiary amine, converting it into a hydrophilic quaternary ammonium salt, which increases Tcp significantly [41]. Despite its effectiveness, pH adjustments show limitations as a control strategy. As shown in Figure 9b, Tcp increased by roughly 15 °C when the pH value decreased from pH 9 to pH 7. Such a sharp and uncontrollable shift is impractical for applications requiring fine-tuned thermoresponsiveness.

**Figure 10:**
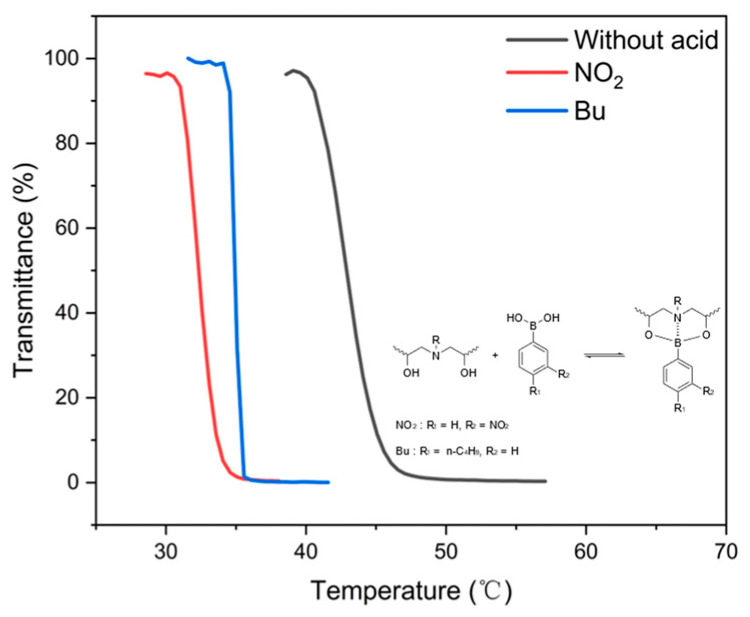
Effects of 3-nitrophenylboronic acid and 4-butylphenylboronic acid additions on the cloud point of C5PEG and the formation of borate esters upon reaction with boric acid, stabilized via intramolecular N-B coordination.

Phenylboronic acid derivatives can react with the diethanolamine (DEA) unit in the main chain of CPEG to form a borate ester and realize boron–nitrogen (B–N) coordination with two five-member rings. This unique coordination enables hydrophobic groups to be anchored to the polymer’s main chain, changing its overall hydrophilicity. Specifically, as shown in Figure 10, the addition of phenylboronic acids with hydrophobic substituents—such as nitro and butyl groups—resulted in notable Tcp reductions, with Tcp values observed at 32.4 °C and 35 °C, respectively. This decrease in Tcp demonstrates that incorporating hydrophobic groups via phenylboronic acid modification effectively enhances polymer hydrophobicity and lowers the transition temperature. These findings indicate that modifying thermoresponsive polymers with different phenylboronic acid derivatives provides a viable strategy for controlling Tcp.

## 4. Conclusions

In this study, a series of novel amphiphilic CPEG copolymers were facilely synthesized through an amine–epoxy click reaction between PEGDE and various primary amines. The chemical structures of CPEGs were confirmed using NMR, ATR-FTIR, and GPC. Detailed characterization confirmed their amphiphilic structures and thermoresponsive behavior via DLS. The transition temperature (Tcp) of the CPEG copolymers was shown to be highly tunable, with a range from 20.8 °C to 46.8 °C achieved by varying side-chain lengths, mixing ratios, salt additions, and phenylboronic acid modifications. While pH adjustments also influenced Tcp, they offered less precision for fine control. These findings highlight the versatility of this CPEG copolymer series for phase-transition temperature regulation, enabling post-synthesis adjustments to Tcp, in which Tcp can be tailored post-synthesis. The wide range of transition temperatures renders them suitable for drug delivery and application in other thermoresponsive medical materials.

## Figures and Tables

**Table 1:** Conditions for and results of the polymerization.

Sample	Aliphatic Amine	Mn (Da) (×10^−3^)	Mw (Da) (×10^−3^)	Ð	Tcp (°C)
C4PEG	*n*-butylamine	6.6	8.3	1.2	46.8
C5PEG	*n*-pentylamine	7.2	9.2	1.3	29.7
C6PEG	*n*-hexylamine	7.1	9.2	1.3	20.8
C5PEG (without solvent)	*n*-pentylamine	13.0	26.4	2.0	-

## Data Availability

Data are contained within the article or Supplementary Materials.

## Supplementary Materials

The following supporting information can be downloaded at https://www.mdpi.com/article/10.3390/polym16243470/s1, Figure S1. (a) GPC curve of C4PEG. (b) GPC curve of C5GPC. (c) GPC curve of C5PEG (without solvent). (d) GPC curve of C6PEG; Figure S2. Absorbance curves of C4PEG solutions at different concentrations across 300–450 nm; Figure S3. Absorbance curves of C6PEG solutions at different concentrations across 300–450 nm; Figure S4. Absorption of the hydrophobic probe DPH in C4PEG and C6PEG copolymer solutions. CMC was determined via extrapolation of the difference of absorbance at 377 and 400 nm. The measurements were carried out at 25 °C; Figure S5. ATR-FTIR spectra of C6PEG and C4PEG across 4000–2500 cm^−1^ and 1900–850 cm^−1^; Figure S6. The impact of different polymer concentrations in solution on Tcp; Figure S7. Repetitive experiments in 25%C4PEG and 75%C4PEG solution.
