# Supplementary material for: Facile Synthesis of Thermoresponsive Alternating Copolymers with Tunable Phase-Transition Temperatures
Source: Polymers (Basel). 2024 Dec 12;16(24):3470. doi: 10.3390/polym16243470 (PMC11728650; doi:10.3390/polym16243470)
Supplement: Supplementary file 1 [file polymers-16-03470-s001.zip › polymers-3317262-supplementary.pdf]

Supplementary Material

Facile Synthesis of Thermoresponsive Alternating Copolymers with Tunable Phase-Transition Temperatures

Zichen Huang [1], Fan Chen [1], Qi Wang [2], Dingxiang Zhang [1], Hongdong Wang [2,*] and Xiacong Zhang [1,*]

1 Department of Polymer Materials, School of Materials Science and Engineering, Shanghai University, Shanghai 200444, China; 18857127216@shu.edu.cn (Z.H.)

2 School of Mechatronic Engineering and Automation, Shanghai University, Shanghai 200444, China

* Correspondence: whd20@shu.edu.cn (H.W.); xczhang@t.shu.edu.cn (X.Z.)

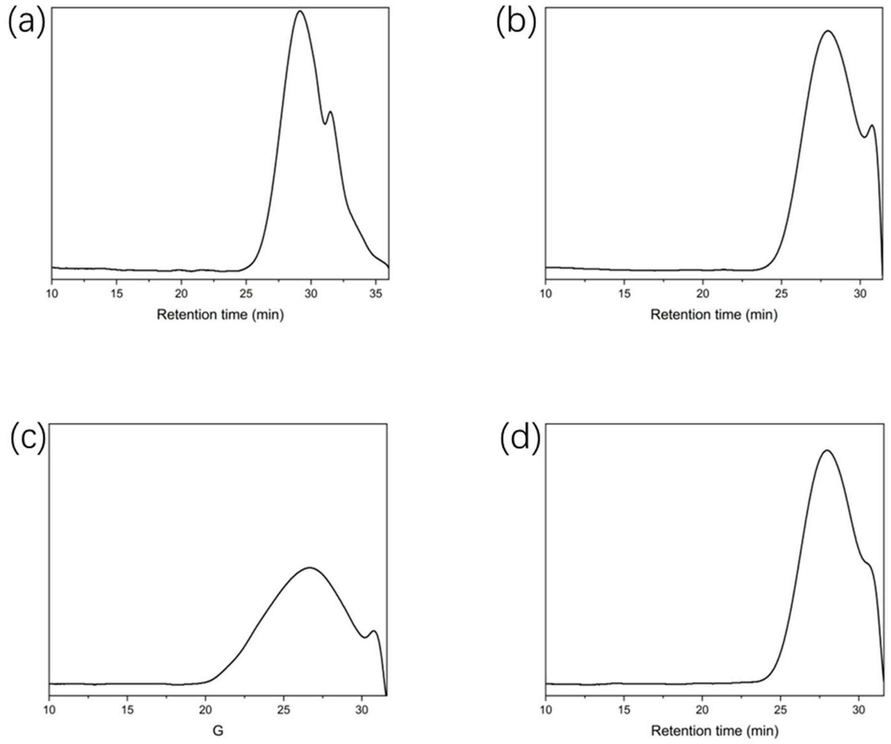

Figure S1. (a) GPC curve of C4PEG. (b) GPC curve of C5GPC. (c) GPC curve of C5PEG (without solvent). (d)GPC curve of C6PEG

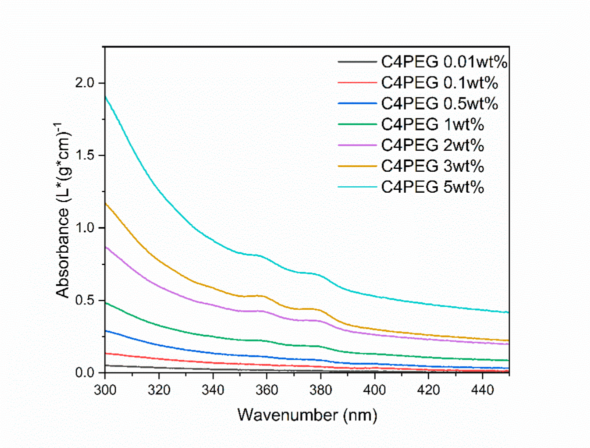

Figure S2. Absorbance curves of C4PEG solutions at different concentrations across 300-450 nm

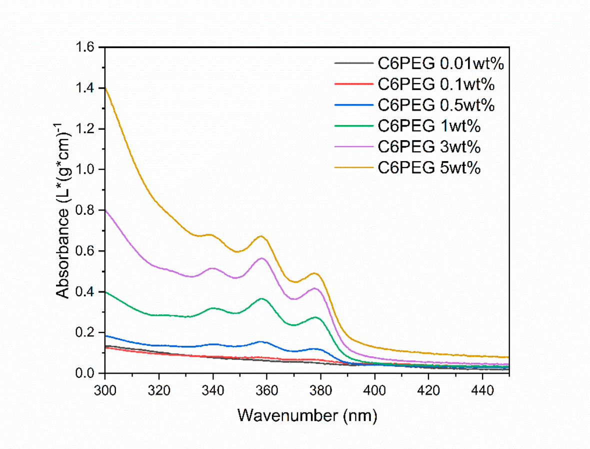

Figure S3. Absorbance curves of C6PEG solutions at different concentrations across 300-450 nm

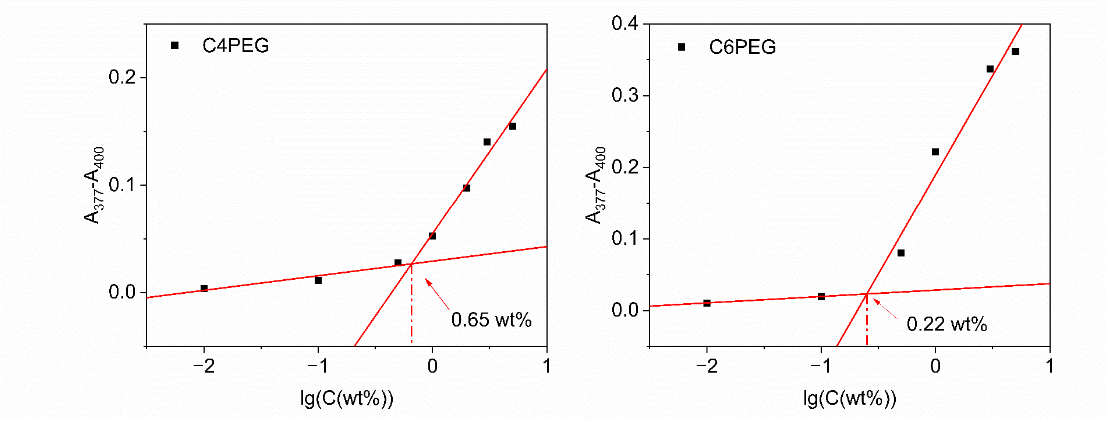

Figure S4. Absorption of the hydrophobic probe DPH in C4PEG and C6PEG copolymer solutions. CMC was determined via extrapolation of the difference of absorbance at 377 and 400 nm. The measurements were carried out at 25 °C.

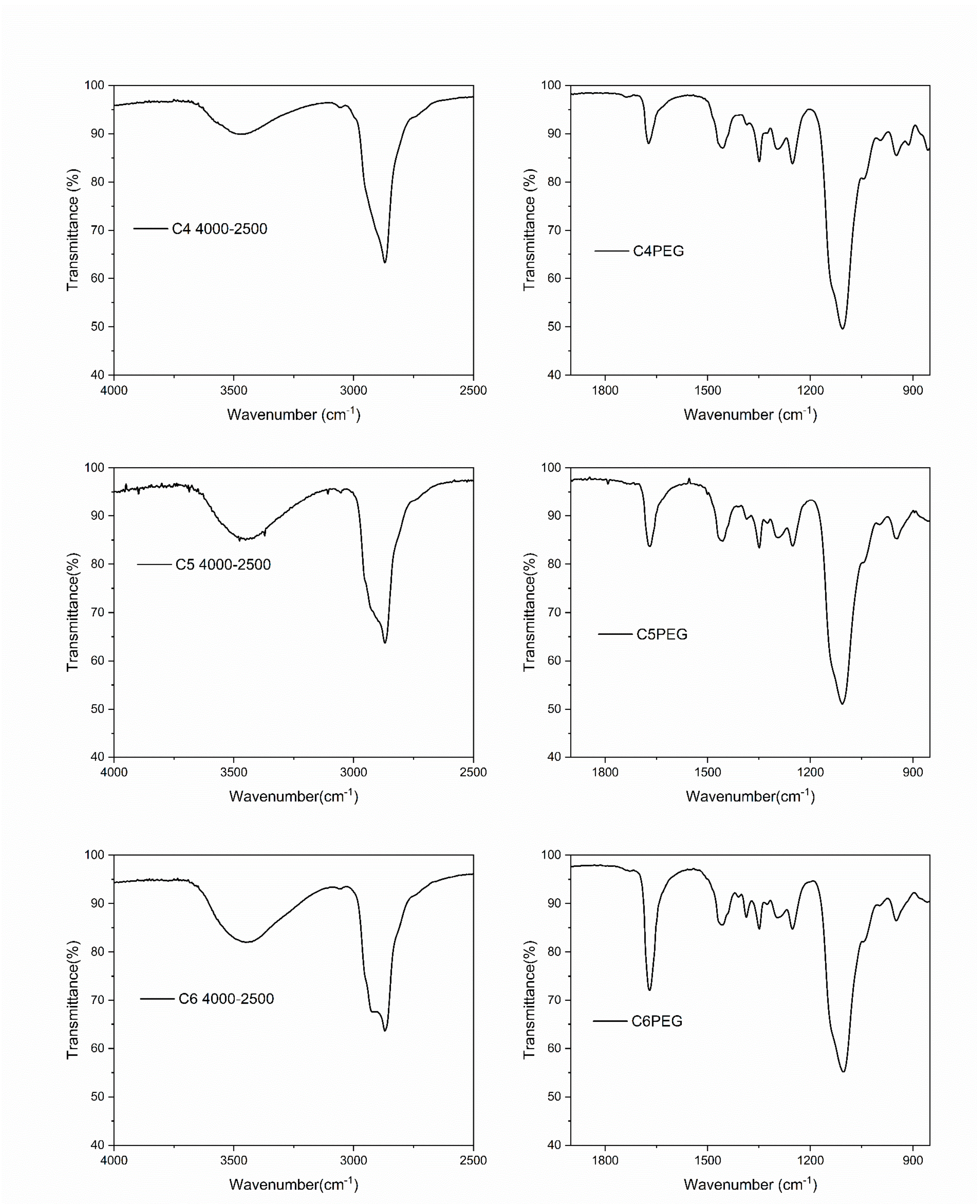

Figure S5. ATR-FTIR spectra of C6PEG and C4PEG across 4000-2500 cm^{-1} and 1900-850 cm^{-1}

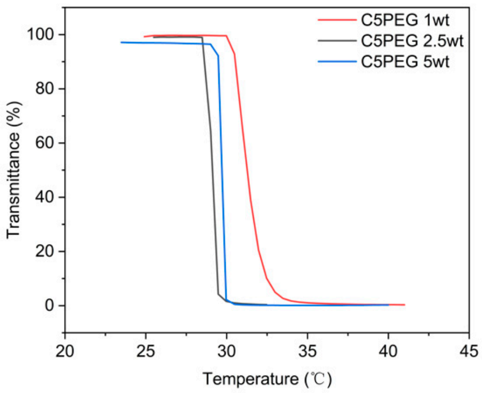

Figure S6. The impact of different polymer concentrations in solution on Tcp

The impact of polymer concentration in solution on Tcp has been studied. However, polymer concentration has small impact on Tcp.

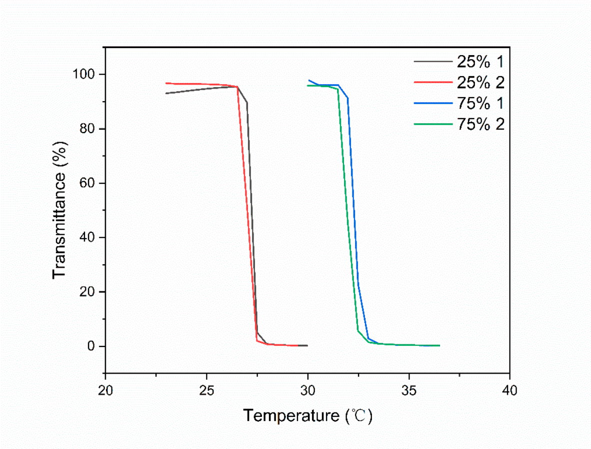

Figure S7. Repetitive experiments in 25%C4PEG and 75%C4PEG solution
